# Recent Advances in Alkaloids from *Papaveraceae* in China: Structural Characteristics and Pharmacological Effects

**DOI:** 10.3390/molecules29163778

**Published:** 2024-08-09

**Authors:** Meixian Zhang, Jing Yang, Yanping Sun, Haixue Kuang

**Affiliations:** Key Laboratory of Basic and Application Research of Beiyao, Heilongjiang University of Chinese Medicine, Ministry of Education, Harbin 150040, China; zhangmeixian58@126.com (M.Z.); mayday111@163.com (J.Y.); sunyanping_1@163.com (Y.S.)

**Keywords:** *Papaveraceae*, alkaloids analysis, pharmacological effect

## Abstract

The *Papaveraceae* plant family serves as a botanical reservoir for a variety of medicinal compounds that have been traditionally utilized in Chinese medicine for numerous generations. Growing attention towards the pharmaceutical potential of *Papaveraceae* has resulted in the identification of many alkaloids, which have attracted significant attention from the scientific community because of their structural complexity and wide range of biological activities, such as analgesic, antihypertensive, antiarrhythmic, anti-inflammatory, antibacterial, anti-tumor, anti-cancer, and other activities, making them potential candidates for medical use. The primary objective of this review is to analyze the existing literature on the historical use of *Papaveraceae* plants, focusing on their alkaloid structures and relationship with pharmacological effects, as well as provide a theoretical basis for their clinical application, with the goal of unveiling the future potential of *Papaveraceae* plants.

## 1. Introduction

When thinking of the *Papaveraceae* family, the opium *poppy* often comes to mind. Rightfully so, as the opium poppy is one of the world’s oldest medicinal plants and remains the only commercial source of narcotic analgesics such as morphine, codeine, and semi-synthetic derivatives like oxycodone and naltrexone [1]. This family includes not only the subfamily *Papaveroideae* but also *Hypecooideae* and *Fumarioideae.* Most plants in this family are herbaceous or slender sub-shrubs, with small shrubs and trees being exceptionally rare. They typically contain milky or pigmented sap. The primary roots are usually slender and fibrous, with tuber formation being infrequent. There are over 700 species in this family, spread across approximately 38 genera. *Corydalis*, the largest genus within the *Papaveraceae* family, consists of numerous species that have been historically used as medicinal plants in East Asia. Among these, *yanhusuo* and *xiatianwu* are particularly well known and are included in the Pharmacopoeia of China [2,3]. *Corydalis* primarily thrives in hilly regions at altitudes ranging from 400 to 1200 m. Notably, more than half of these species are found in China, with over 360 species distributed across 18 genera. The group includes genera such as *Corydalis*, *Chelidonium*, *Dactylicapnos*, *Dicranostigma*, *Eomecon*, *Hypecoum*, *Macleaya*, *Meconopsis*, *Papaver*, *Hylomecon*, *Adlumia*, *Argemone*, *Dicentra*, *Eschscholtzia*, *Fumaria*, *Glaucium*, *Roemeria*, and *Stylophorum*. To date, only the first ten genera have been extensively studied. It is also worth noting that *eomecon* is a species native to China. Chinese poppy plants are primarily found in the southwestern part of the country, particularly in genera such as *Corydalis* and *Meconopsis*. This region also hosts a limited range of *Papaver* and *Eschscholtzia* species, in addition to a few species like *Hylomecon*, *Chelidonium*, *Macleaya*, and *Hypecoum*, which are widely distributed throughout China. The predominant occurrence of *Papaveraceae* flora in China is concentrated in the southwestern part of the country, as indicated by reference [4]. Several medicinal plants from the poppy family are listed in the Chinese Pharmacopoeia, including *Chelidonii herba*, *Corydalis rhizoma*, and *Corydalis decumbentis rhizoma*. *Papaveraceae* plants primarily comprise alkaloids, which exhibit notable pharmacological properties. Additionally, they are used conventionally for cultivating decorative flora and incorporating herbs and seasonings in culinary practices [5,6,7,8,9]. Besides alkaloids, these plants also contain flavonoids, volatile oils, furan compounds, and various non-alkaloid constituents [10]. Recently, significant progress has been made in the study of alkaloids from *Papaveraceae*, attributed to ongoing advancements in science and technology and the refinement of research methodologies. Most articles focus on individual plant species or genera, providing limited coverage of poppy alkaloids as a whole. The objective of this review is to provide a detailed analysis of alkaloids from *Papaveraceae*, focusing on structure characteristics, associated pharmacological activities, and method of detection. Using the keywords “*Papaveraceae*”, “alkaloid”, “biological activity of alkaloid”, and “detection”, a comprehensive review of the literature from the past two decades was conducted across multiple databases, including PubMed, Web of Science, China National Knowledge Infrastructure (CNKI), and Google Scholar. The articles were gathered and organized based on their respective topics before being integrated into the review. Some information from the last 40 years was also included.

## 2. Alkaloids

**Figure 1:**
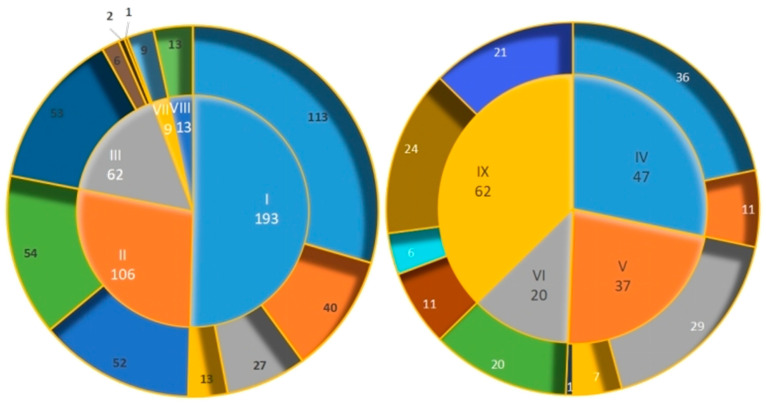
The number of various alkaloids in the *papaver* family. I: benzophenanthridine (including dihydrobenzo[c]phenanthridines—113, hexahydrobenzo[c]phenanthridine—40, quaternary benzophenanthridine—13, and dimeric dihydrobenzophenanthridines—27); II: protoberberine (including berberines—52 and protoberberines—54); III: aporphine (including aporphines—53, proaporphines—6, isoproaporphines—2, and aporphine dimers—1); IV: benzylisoquinoline (including 1-benzylisoquinolines—36 and spirobenzylisoquinolines—11); V: benphthaleoquinoline (including phthaloisoquinolines—29, cleavage-ring phthaloisoquinolines—7, and dimeric cleavage-ring phthaloisoquinolines—1); VI: simple isoquinoline (20); VII: protopine (9); VIII: morphine (13); and IX: others (62).

Alkaloids, known for their diverse biological activities such as analgesic, antihypertensive, anti-tumor, and hepatoprotective effects, are integral to our understanding of the therapeutic potential of this family. Over 540 alkaloid compounds have been identified in plants belonging to the *Papaveraceae* family (Table S1). These compounds are widely acknowledged as significant bioactive constituents in plant species. The category of alkaloids being examined primarily consists of isoquinoline alkaloids, characterized by their fundamental isoquinoline structure [11,12,13,14,15,16,17,18,19,20,21,22,23,24,25,26,27,28,29,30,31,32,33,34,35,36,37,38,39,40,41,42,43,44,45,46,47,48,49,50,51,52,53,54,55,56,57,58,59,60,61]. Isoquinoline alkaloids are categorized into different groups based on their unique nuclear parent structures, including benzophenanthridine (I **1~193**), protoberberine (II **194~299**), aporphine (III **300~361**), benzylisoquinoline (IV **362~408**), benphthaleoquinoline (V **409~445**), simple isoquinoline (VI **446~465**), protopine (VII **466~474**), and morphine (VIII **475~487**). Additionally, 62 other alkaloids have been isolated and identified from the *Papaveraceae* family (IX **488~549**). Among these, benzophenanthridine alkaloids are the most prevalent, accounting for 35% of known alkaloids. By contrast, protopine alkaloids are the least common, with only nine identified from the poppy family (Figure 1).

**Figure 2:**
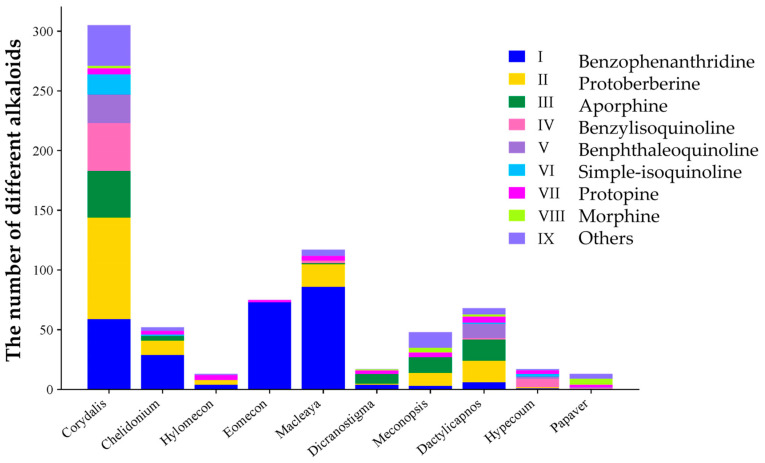
The known alkaloids in different genera.

The findings of this research indicated that *Corydalis* exhibited the greatest abundance of alkaloids, comprising a sum of 305 compounds representing 55% of the overall alkaloid content. This finding can be explained by the high prevalence of alkaloid-rich compounds in the plant. *Macleaya* demonstrated the second-highest concentration of alkaloids, while *Papaver* exhibited the lowest level, with only 13 alkaloids being isolated and characterized (Figure 2).

### 2.1. Benzophenanthridine Alkaloids

**Figure 3:**
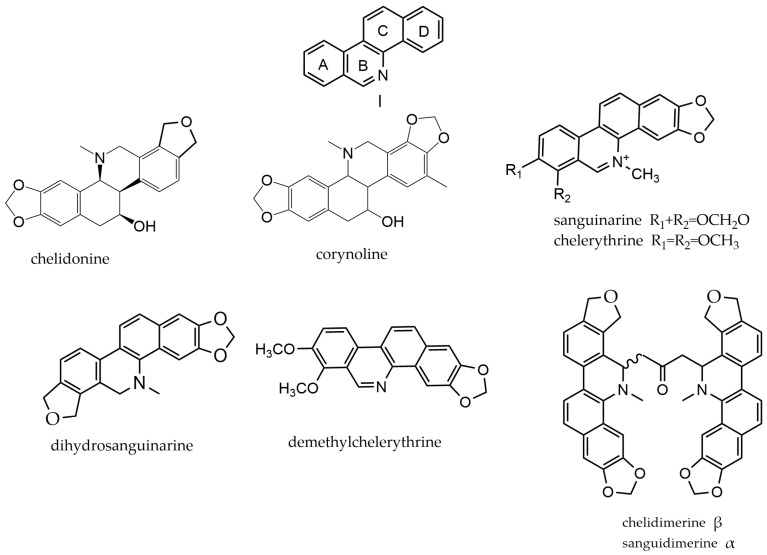
Benzophenanthridine alkaloids’ parent nucleus and representative compounds. I parent nucleus of benzophenanthridine alkaloids; chelidonine and corynoline are type of I-i; sanguinarine and chelerythrineand are type of I-ii; dihydrosanguinarine and demethylchelerythrine are type of I-iii; and chelidimerine and sanguidimerine are type of I-iv.

Benzophenanthridine alkaloids consist of four six-membered rings (A, B, C, and D) that are parallel to each other. The A and D rings are aromatic, while the B and C rings contain both aromatic and hydrogenated aromatic sections. Typically, there are two oxygen-containing substituents on the non-azido ring, mostly methoxy or hydroxy groups. The compounds (**1**–**193**) can be classified into hexahydrobenzo[c]phenanthridine (I-i), quaternary benzophenanthridine alkaloids (I-ii), and dihydrobenzo[c]phenanthridines (I-iii) based on the level of hydrogenation in the B and C rings. Dihydrobenzo[c]phenanthridines can also polymerize to form dimeric dihydrobenzophenanthridines (I-iv). In types I-i and I-iii, the N atom is usually demethylated. Ultimately, the quaternary benzophenanthridine alkaloids result from the N protonation of types I-i or I-iii, leading to the corresponding ammonium quaternary salts [62]. The parent nucleus and representative compounds of benzophenanthridine alkaloids are shown in Figure 3.

These compounds are predominantly found in *Macleaya*, *Eomecon*, *Corydalis*, and *Chelidonium*. *Eomecon* yielded a total of 75 compounds, out of which 73 were identified as benzophenanthridine alkaloids. Interestingly, only one alkaloid of this class has been found in *Hypecoum*, which is bungeanine.

### 2.2. Protoberberine Alkaloids

**Figure 4:**
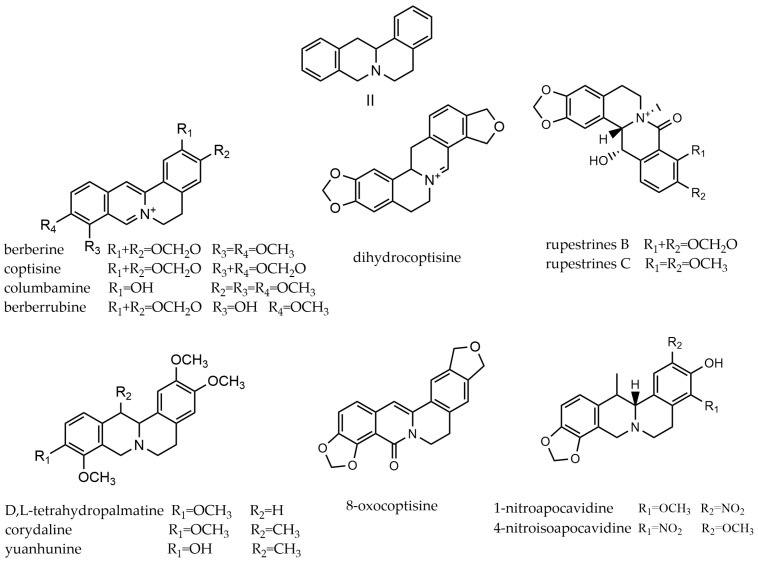
Protoberberine alkaloids’ parent nucleus and the representative compounds. II parent nucleus of protoberberine alkaloids; berberine, coptisine, columbamine, berberrubine, dihydrocoptisine, rupestrines B, and rupestrines C are type of II-i; and corydaline, d,l-tetrahydropalmatine, yuanhunine, 8-oxocoptisine, 1-nitroapocavidine, 4-nitroisoapocavidine are type of II-ii.

Protoberberine alkaloids consist of two isoquinoline rings, as based on the dibenzo[a,g]quinolizidine system. These tetracyclic alkaloids are derived from benzylisoquinolines through phenolic oxidation and coupling with the isoquinoline n-methyl group, which becomes the “berberine bridge” carbon [63]. The C-1, C-2, C-3, C-9, C-10, and C-11 of these compounds are often linked with hydroxyl, methoxy, nitro, or other substituents. Usually, C-1 and C-2, C-2 and C-3, C-9 and C-10, or C-10 and C-11 form a five-membered oxygen ring through a methylenedioxy group. C-13 and C-14 have two configurations of α and β [64]. The compounds (**194**~**299**) can be classified into two types based on the degree of D-ring oxidation in its structure: berberines (II-i) and protoberberines (II-ii). Berberine is mostly composed of quaternary ammonium bases, found in plants such as *Chelidonium majus* L. and *Corydalis edulis* Maxim. Protoberberines, mostly composed of tertiary amine bases, such as the d,l-tetrahydropalmatine found in Dongbei *yanhusuo*. Protoberberine alkaloids parent nucleus and the representative compounds are shown in Figure 4.

These kinds of alkaloids are primarily present in *Corydalis*, *Macleaya*, and *Dactylicapnos*. Only one of these alkaloids was found in both *Hypecoum* and *Dicranostigma*, such as coptisine and isocorypalmine, but not in *eomecon* and *papaver*.

### 2.3. Aporphine Alkaloids

**Figure 5:**
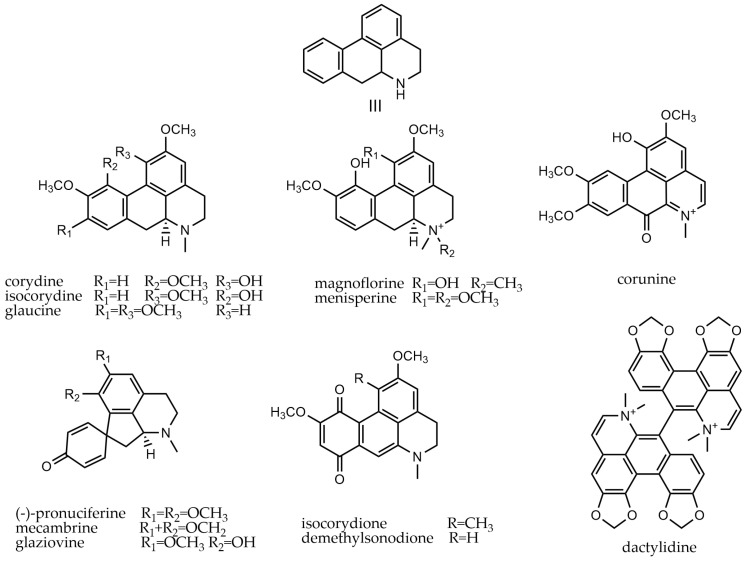
Aporphine alkaloids’ parent nucleus and the representative compounds. III parent nucleus of aporphine alkaloids; corydine, isocorydine, glaucine, magnoflorine, menisperine, corunine are type of III-i; (-)-pronuciferine, mecambrine, glaziovine are type of III-ii; isocorydione and demethylsonodione are type of III-iii; and dactylidine are type of III-iv.

Aporphine alkaloids, characterized by a heterocyclic aromatic basic skeleton, are known to originate from various organisms [65]. The C ring of aporphine alkaloids shares three carbon atoms with the isoquinoline structure. The aromatic ring substituents (primarily H, OH, OCH_3_, -CH_2_O-), nitrogen substituents (H, OH, CH_3_, COCH_3_, etc.), and variations in R and S configurations result in a rich diversity of structural types within this class of compounds. These alkaloids, ranging from 194 to 361, can be classified into aporphines (III-i), proaporphines (III-ii), isoproaporphines (III-iii), and aporphine dimers (III-iv). Proaporphines are typically considered precursors to aporphines. Isoproaporphines, which contain two ketone groups, are rare, with only two compounds—namely isocorydione and demethylsonodione—having been isolated and identified. Currently, only dactylidine, an aporphine dimer, has been isolated from *dactylicapnos*. The parent nucleus of aporphine alkaloids and their representative compounds are depicted in Figure 5.

Aporphine alkaloids are widely found in the genera *Corydalis*, *Meconopsis*, and *Dactylicapnos*. By contrast, only four compounds of this class have been identified in *Chelidonii Herba*. However, they have not been found in the genera *Hylomecon*, *Eomecon*, and *Papaver*.

### 2.4. Benzylisoquinoline Alkaloids

**Figure 6:**
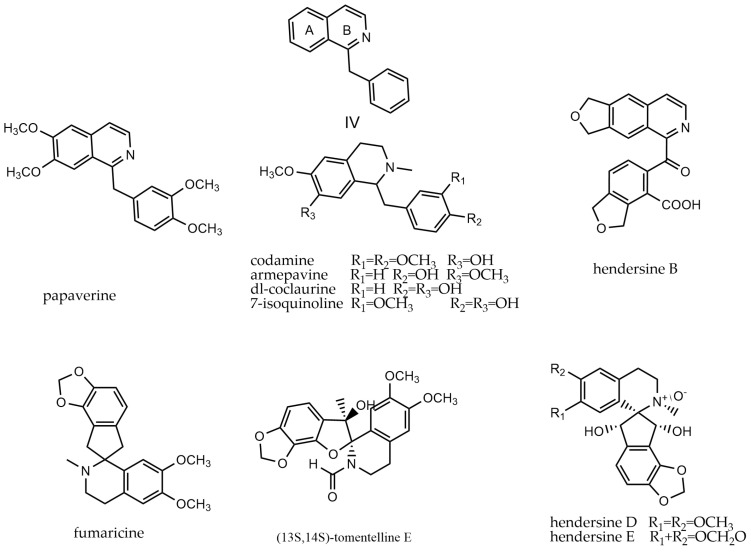
Benzylisoquinoline alkaloids’ parent nucleus and the representative compounds. IV parent nucleus of benzylisoquinoline alkaloids; papaverine, codamine, armepavine, dl-coclaurine, 7-isoquinoline, hendersine B are type of IV-i; and fumaricine, (13*S*,14*S*)-tomentelline E, hendersine D, hendersine E are type of IV-ii.

The benzylisoquinoline structure serves as the foundational framework for numerous alkaloids exhibiting diverse structural characteristics. It is a heterocyclic aromatic organic compound and a structural isomer of quinoline [66]. This group of alkaloids (**362**–**408**) is characterized by an isoquinoline skeleton and can be divided into 1-benzylisoquinolines (IV-i) and spirobenzylisoquinolines (IV-ii). 1-Benzylisoquinolines are derivatives of isoquinoline with a benzyl group attached to the 1-position of the parent nucleus. An example of this type of compound is papaverine. On the other hand, spirobenzylisoquinolines are derivatives of isoquinoline where the B and C rings form a spirocycle. An example of this type of compound is fumaricine. The parent nucleus of benzylisoquinoline alkaloids and representative compounds are shown in Figure 6.

The *Papaveraceae* family of plants has yielded a total of 47 benzylisoquinoline alkaloids. These alkaloids are more commonly found in *Corydalis*, with seven identified in *Hypecoum* and two in *Macleaya*. *Papaver* and *Dactylicapnos* each contain one, specifically papaverine and (*R*)-magnocurarine. Other genera do not contain any benzylisoquinoline alkaloids.

### 2.5. Benphthaleoquinoline Alkaloids

**Figure 7:**
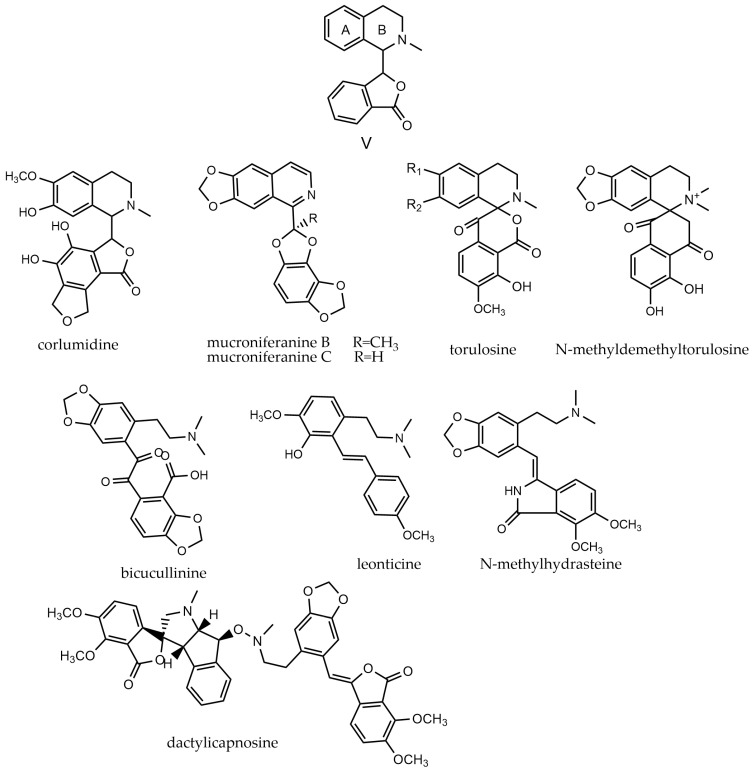
Benphthaleoquinoline alkaloids’ parent nucleus and the representative compounds. V parent nucleus of benphthaleoquinoline alkaloids; corlumidine, mucroniferanine B, mucroniferanine C, torulosine, *N*-methyldemethyltorulosine are type of V-i; bicucullinine, leonticine, *N*-methylhydrasteine are type of V-ii, dactylicapnosine is type of V-iii.

This class of alkaloids comprises isoquinoline and phthalide fragments connected at the C-1 position. The B ring of isoquinoline may exist in a ring-opened form, often featuring a dimethylaminoethyl side chain [64]. These alkaloids (**409**–**445**) are categorized into three groups based on the structure of the B-ring: phthaloisoquinolines (V-i), cleavage-ring phthaloisoquinolines (V-ii), and dimeric cleavage-ring phthaloisoquinolines (V-iii). The V-i type B rings primarily consist of six-membered rings, though a few five-membered rings, such as torulosinine, also exist. The V-ii type B rings, found in ring-opened compounds like leonticine, exhibit distinct structures. Type V-iii involves the dimerization of two benzelisoquinolines, with only one known example in the *Papaveraceae* family, namely dactylicapnosine. The parent nucleus of benphthaleoquinoline alkaloids and representative compounds are depicted in Figure 7.

A total of 37 phthaloisoquinolines have been isolated from plants of the *Papaveraceae* family. *Corydalis*, *Dactylicapnos*, and *Papaver* are the sole genera where these alkaloids have been identified, with *Corydalis* being the most prevalent. *Dactylicapnos* has isolated and identified two compounds of this class, namely torulosine and *N*-methyldemethyltorulosine.

### 2.6. Simple-Isoquinoline Alkaloids

**Figure 8:**
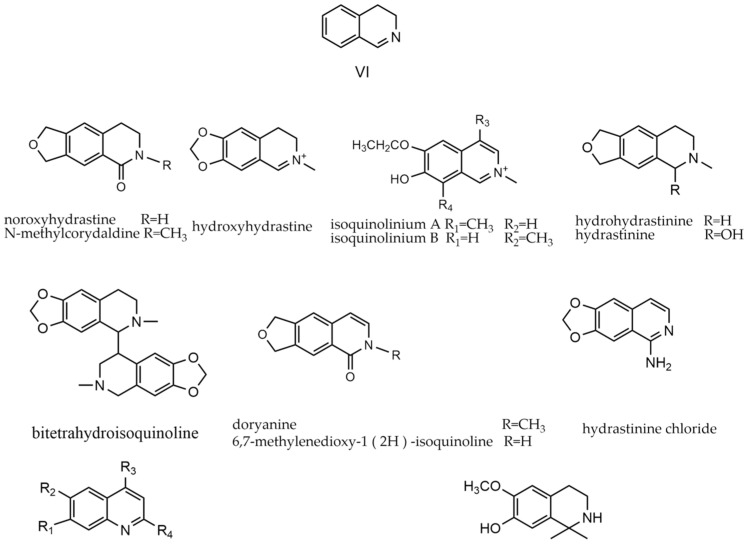
Simple-isoquinoline alkaloids’ parent nucleus and the representative compounds. VI parent nucleus of simple-isoquinoline alkaloids.

Simple isoquinoline is a natural substrate for the biosynthesis of protoberberine-type and aporphine-type alkaloids. The substituents resemble those found in protoberberine types [67]. This class of alkaloids (**446**–**465**) consists solely of the isoquinoline skeleton as the parent nucleus. Such alkaloids can also form quaternary ammonium bases, such as hydroxyhydrastine. The parent nucleus of simple-isoquinoline alkaloids and representative compounds are depicted in Figure 8.

There are relatively few alkaloids in this class, with only 20 having been isolated and identified. These are mainly found in the genera *Corydalis*, with two in *hypecoum* and one in *Chelidonium* and *Dactylicapnos*. No alkaloids belonging to this class have been discovered in any other genus.

### 2.7. Protopine Alkaloids

**Figure 9:**
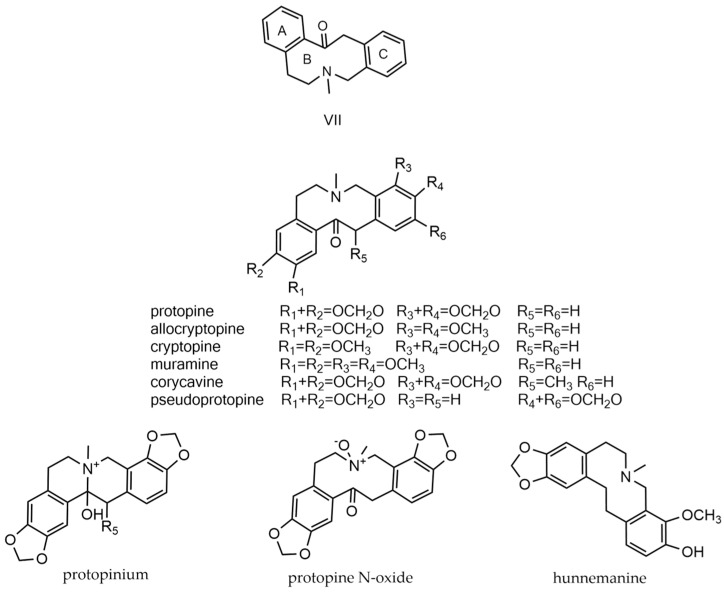
Protopine alkaloids’ parent nucleus and the compounds. VII parent nucleus of protopine alkaloids.

Protopine alkaloids constitute a class of natural isoquinoline compounds characterized by a basic three-ring structure, namely two benzene rings (A and C rings) and a nitrogen heterocyclic ring with ten elements (B ring) [68]. Most structures within this class feature oxygen groups (-OCH_3_, -OCH_2_O) located at positions C2–C3 of ring A and C9–C10 of ring C; in rarer cases, such as pseudoprotopine, the oxygen group is situated at position C10–C11 of the C ring. The structures of protopine alkaloids (**466**–**474**) typically include tertiary and quaternary amine functionalities, the specific forms of which are influenced by the pH of the environment. Studies have identified protopinium, protopine N-oxide, and hunnemanine as metabolites of protopine alkaloids [69,70,71,72,73]. Figure 9 illustrates the parent nucleus of protopine alkaloids and representative compounds.

Nine compounds from this class have been isolated from the poppy family. Despite their limited number, alkaloids of this class are found across various genera.

### 2.8. Morphine Alkaloids

**Figure 10:**
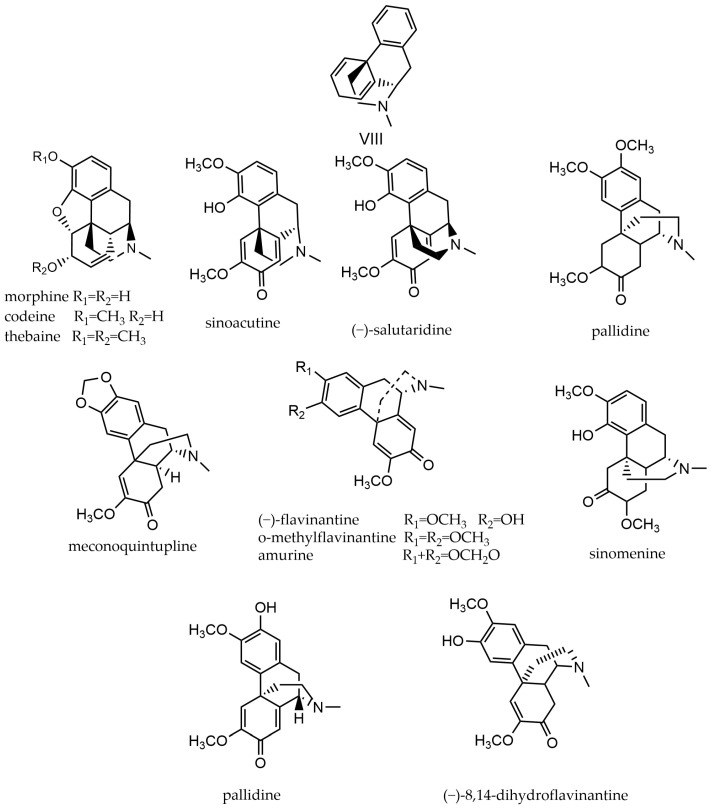
Morphine alkaloids’ parent nucleus and the compounds. VIII parent nucleus of morphine alkaloids.

Morphine alkaloids, which are derivatives of benzylisoquinoline that are partially saturated phenanthrene derivatives. Naturally occurring morphinanes are complex, molecules with intricate stereostructures, consisting of five rings. They include a five-membered dihydrofuran ring, a nitrogen-containing bridging ring, and five consecutive chiral centers, one of which is a quaternary carbon center at the benzyl position [74]. Morphine and codeine were commonly used as initial substrates for producing pharmacologically beneficial compounds on an industrial scale until thebaine became more readily accessible [75]. The parent nucleus of morphine alkaloids and their compounds are shown in Figure 10.

Thirteen morphinan alkaloids (**475**–**487**) have been identified and isolated from the poppy family. These alkaloids are primarily found in *Papaver* and *Dactylicapnos*, with small amounts also present in *Corydalis*.

### 2.9. Other Alkaloids

**Figure 11:**
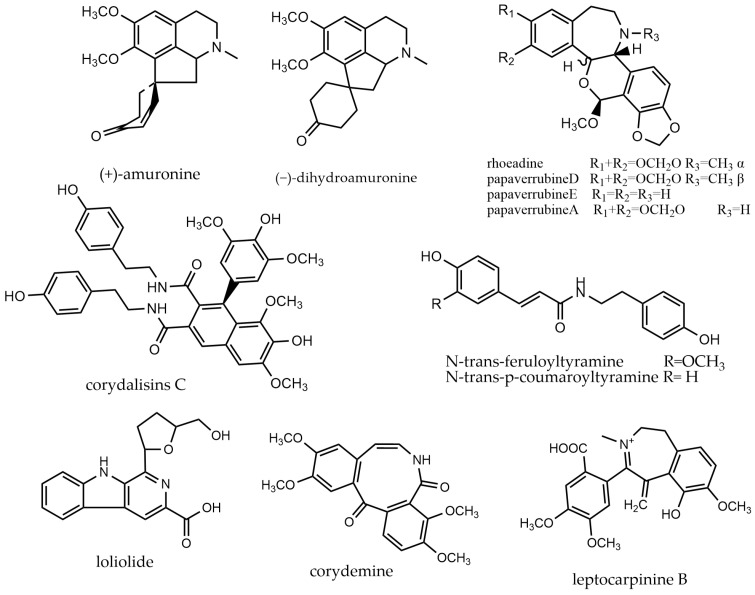
Other alkaloids and their representative compounds.

In addition to the previously mentioned isoquinoline alkaloids, the poppy family also contains other types such as isoquinoline alkaloids (**488**–**498**) and lichondine alkaloids (**499**–**504**), characterized by a benzene ring and a heptane ring structure. Furthermore, there are organic amines (**505**–**528**) that lack nitrogen atoms in the ring, along with additional alkaloids (**529**–**549**), as illustrated in Figure 11.

These alkaloids are predominantly found in *Corydalis*, *Meconopsis*, *Chelidonium*, *Macleaya*, *Papaver*, and *Dactylicapnos*, with a small number also occurring in *Hypecoum* and *Hylomecon*. Examples include zarzissine [35] and leptocarpinine B [34]. No other classes of alkaloids were identified in *Dicranostigma*.

## 3. Pharmacological Actions and Clinical Applications

**Figure 12:**
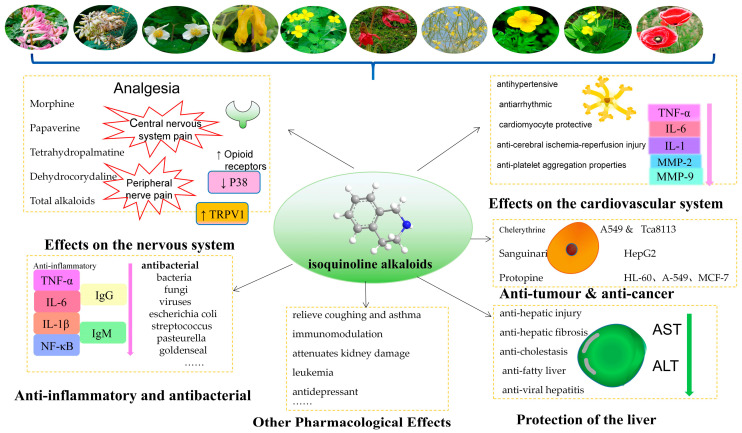
The pharmacological effects of *Papaveraceae*.

The poppy plant has been found to exert pharmacological effects on both the nervous and cardiovascular systems. It has demonstrated effectiveness in treating coughs and asthma, in addition to possessing antibacterial and antidiarrheal properties. Recent research has further unveiled its anti-inflammatory, antioxidant, anti-fibrotic, and anti-tumor properties, suggesting potential utility in treating diabetes mellitus. Figure 12 illustrates some alkaloids and pharmacological effects of *Papaveraceae*.

The pharmacological effects of alkaloids derived from *Papaveraceae* exhibit variability based on the specific extraction technique employed. *Yanhusuo*, a traditional medicine from the *Papaveraceae*, is typically processed using vinegar. The production of vinegar has been found to lead to higher levels of protopine (**466**) and tetrahydrocoptisine (**198**). At the same time, its analgesic effect was enhanced, while the levels of berberine (**196**), palmatine (**207**) and dehydrocorydaline (**266**) are notably reduced following vinegar processing [76]. Research findings suggest that the total alkaloid of *Corydalis rhizoma* can be extracted using a process involving 70% ethanol refluxing, followed by acidification with 20% HCl and alkalization with 5 M NaOH. These total alkaloids have been shown to inhibit thrombin-induced platelet aggregation [77]. In addition, the extraction of *Chelidonium majus* using 80% methanol has been found to result in the highest total alkaloid content and exhibit significant antibacterial properties. Furthermore, the extraction of benzophenanthridine alkaloids from *Macleaya cordata (Willd) R.* with potential anti-cancer properties was successfully achieved through ultrahigh pressure extraction and pH-zone-refining counter-current chromatography [78,79].

### 3.1. Effects on the Nervous System

The effect of *Papaveraceae* alkaloids on the nervous system primarily manifests through their analgesic properties. As technology progresses, an increasing number of alkaloid medications possessing analgesic properties are being identified. Studies have shown that the alkaloids found in the *Papaveraceae*, including morphine alkaloids protoberberine alkaloids, benzophenanthridine alkaloids, and aporphine alkaloids et al. possess analgesic properties.

Morphine (**475**) is a potent opioid analgesic, is renowned for its efficacy in pain relief, and is commonly employed for the management of cancer-related pain since the 1950s [80]. Tetrahydropalmatine (DL-THP) (**219**), as a traditional analgesic agent, has been used for the treatment of neuropathic and inflammatory mild-to-moderate pain in China. It exhibits both central dopamine (DA) presynaptic and postsynaptic receptor-blocking effects and has been suggested as a potential alternative to opioid analgesics [80,81,82,83]. Dehydrocorydaline (DHC) (**266**) demonstrated efficacy in alleviating pain induced by complete Freund’s adjuvants (CFAs), acetic acid, and formalin in mice. In these investigations, naloxone was observed to diminish the antinociceptive effects of DHC, suggesting the involvement of the opioid system in the analgesic mechanism of action of DHC [80,84]. However, its analogous compound, dehydrocorybulbine (DHCB) (**216**), demonstrated efficacy in inhibiting reactions to chemically induced, inflammation-derived, and injury-induced pain, and it exhibited antagonistic effects on D2 receptors [85]. Berberine (**196**), a compound present in diverse plants, demonstrates notable analgesic characteristics. Research indicates that its analgesic effects involve actions such as antagonizing mu opioid receptors (MORs) and delta opioid receptors (DORs), alleviating neuropathic pain through the modulation of TRPV1 expression and exhibiting anti-cholinesterase activity [86,87,88]. Additionally, berberine’s impact on the endogenous opioid system is attributed to its capacity to readily penetrate the blood–brain barrier [89]. Moreover, some alkaloids’ analgesic mechanisms have not been fully determined; for instance, palmatine (**207**) and magnoflorin (**300**) have the potential to induce analgesia by modulating anti-inflammatory pathways [90,91]. Columbamine (**204**) has the potential to induce analgesia by inhibiting the enzyme monoamine oxidase B [92].

Additionally, there are documented reports of the analgesic effects of the total alkaloids found in the Papaveraceae family. It has been found that the total alkaloids of *Yanhuanglian* may have a positive impact on alleviating cisplatin-induced peripheral neuropathic pain. On one hand, this effect is related to the inhibition of neuronal damage and the loss of nerve fibers in the epidermis caused by the inflammatory response. On the other hand, it is associated with the inhibition of p38 phosphorylation induced by inflammatory factors and the activation of its downstream TRPV1 receptor [93]. Furthermore, the total alkaloid extract of the congener *Corydalis* impatiens was found to have an obvious analgesic effect on pain induced by acetic acid-induced writhing reaction in mice [94]. A published paper has demonstrated that the analgesic effect of *Chelidonii herba* after vinegar processing is better than that of raw *Chelidonii herba* products for both peripheral pain (acetic acid-induced pain) and central pain (hot plate-induced pain) models in mice. Additionally, the analgesic effect shows a positive correlation with dosage [95]. Moreover, the n-butanol extract of *Hypecoum erectum* L. exhibits a certain analgesic effect [96]. Intramuscular injection of *Corydalis decumbentis rhizoma* can effectively reduce pain in patients undergoing adjuvant treatment for postherpetic neuralgia (PHN), without increasing the burden on liver and kidney functions or causing adverse reactions. The mechanism may be related to the promotion of β-endorphin release and inhibition of plasma substance P release [97]. *Meconopsis quintuplinervia Reg.* contains both total flavonoids and total alkaloids, which have shown significant analgesic effects. It has been observed that the analgesic inhibitory effect of total alkaloids is superior to that of total flavonoids [98]. Furthermore, research has demonstrated that alkaloids found in *Corydalis yanhusuo* possess properties that have anti-addiction, anxiolytic, anti-depressant, anti-Alzheimer’s disease, and anti-epileptic effects. These effects are primarily achieved through the regulation of neurotransmitters, the hypothalamic–pituitary–adrenal (HPA) axis, nitric oxide (NO) production, inflammation, and various signaling pathways, among other mechanisms [99].

### 3.2. The Importance of the Cardiovascular System

The alkaloids of *Papaveraceae* have been found to exert a positive impact on the cardiovascular system, offering potential benefits such as antihypertensive, antiarrhythmic, cardiomyocyte protective, anti-cerebral ischemia–reperfusion injury, and anti-platelet aggregation properties.

Most research in this field has focused on protoberberine alkaloids and total alkaloids, with some studies also addressing protopine alkaloids and benzophenanthridine alkaloids. Notably, all protoberberine alkaloids demonstrate anti-inflammatory properties. One study showed that DL-THP (**219**) helped protect the heart muscle from damage caused by ischemia/reperfusion and decreased cell death and inflammation by activating the PI3K/Akt pathway [100]. Palmatine (**207**) has been documented to mitigate myocardial ischemia/reperfusion injury by inhibiting oxidative stress and the inflammatory response [101]. DHC (**266**) was observed to alleviate myocardial ischemia/reperfusion damage by suppressing apoptosis, inflammation, and oxidative stress through the inhibition of the TNF receptor-associated factor 6 (TRAF6)/NF-κB signaling pathway [102]. Moreover, dehydrocorydaline (**266**) has demonstrated effectiveness in regulating the progression of atherosclerosis in ApoE-deficient mice, resulting in improved aortic compliance, enhanced plaque stability, and reduced systemic and vascular inflammatory responses [103]. Berberine (**196**) was found to activate the Silent Information Regulator-1 (SIRT1) signaling pathway following myocardial ischemia/reperfusion, as well as the Janus kinase 2/signal transducer and activator of transcription (JAK2/STAT3) signaling pathway, leading to reduced apoptosis and oxidative stress. Additionally, Berberine increased the level of miR-26b-5p to inhibit the COX-2/MAPK pathway, thereby restoring cardiomyocyte viability [104,105,106]. DHC (**266**) and tetrahydroberberine (THB) (**205**) demonstrate anti-platelet aggregation properties. DHC is believed to influence ADP receptors P2Y1 and P2Y12, while THB is thought to interact with the THR receptor protease-activated receptor-1 (PAR1) to modulate the downstream Gi/PI3K pathway [107]. It is noteworthy that protopine alkaloids have demonstrated significant efficacy in anti-cerebral ischemia/reperfusion injury and cardiac arrhythmia. Protopine (**466**) is able to produce effective protection for injury caused by focal cerebral ischemia in rats, possibly through the multiple effects of calcium antagonism, antioxidation, and depression of cell apoptosis. It has also been found that intravenous administration of protopine (**466**) at concentrations of 1 mg/L and 2 mg/L can induce the vasodilation of vascular smooth muscle and reduce peripheral resistance, potentially leading to antihypertensive effects [108,109]. Allocryptopine (**467**) has been found to offer substantial protection against experimental arrhythmias induced by various factors such as increased doses of premature ventricular contractions, ventricular tachycardia, ventricular fibrillation, and arrest induced by chloroform, CaCl_2_-ACh mixture, and epinephrine; the process may involve diminishing the self-regulation of the rats’ isolated atrium and papillary muscles [110]. Chelerythrine (**3**) has been shown to help reduce myocardial injury caused by renal ischemia/reperfusion by regulating the protein kinase C/NF-κB pathway to activate CSE/H2S [111].

Numerous studies have been conducted to investigate the impact of total alkaloids on the cardiovascular system. Total alkaloids of *Corydalis rhizoma* specifically demonstrates various pharmacological effects on the cardiovascular system, including improvement in hemorheology, anti-atherosclerosis, anti-myocardial ischemia and reperfusion injury, anti-ventricular remodeling, anti-arrhythmia, anti-hypertension, and anti-thrombosis, among others [112]. Studies have observed that *Corydalis rhizoma* extract has the potential to increase plasma viscosity in rats with blood stasis [103]. *Corydalis decumbentis rhizoma* injection has been found to protect against cerebral ischemic injury by reducing acetylcholinesterase activity in brain tissue, potentially through neuronal protection and anti-neuronal apoptosis, without affecting ICAM-1 mRNA levels [113,114]. Research indicates that *Corydalis decumbentis rhizoma* tablets possess a superior anti-arrhythmic effect. Furthermore, they have been observed to improve neurological function and blood rheological indexes in patients with blood stasis during the acute stage of cerebral infarction [115,116]. Additionally, the total alkaloids from *Corydalis decumbentis rhizoma* have been found to effectively inhibit platelet aggregation induced by shear force [117]. The total alkaloids present in *Corydalis adunca maxim* have shown potential effects in reducing blood pressure and inhibiting the vasoconstrictive effects induced by norepinephrine. Furthermore, intravenous injection of *Corydalis* general alkaloids has been demonstrated to significantly decrease arterial blood pressure while potentially stimulating heart function and improving cardiac contractile function [118,119]. The study indicates that the 85% ethanol extract of *Corydalis* hendersonii exhibits potential anti-inflammatory and anti-platelet effects. These findings underscore the extract’s potential as a therapeutic agent for inflammatory and cardiovascular diseases. The extract significantly reduces the expression of TNF-α, IL-6, and IL-1β inflammatory factors in mouse plasma, as well as the production of MMP-2 and MMP-9 proteins in post-ischemic myocardium. Moreover, the extract significantly inhibits platelet aggregation induced by thrombin, adenosine-5-diphosphate, and arachidonic acid, comparable to aspirin. These results suggest that the extract of *Corydalis* hendersonii holds promise as a therapeutic option for treating inflammatory and cardiovascular diseases [120].

### 3.3. Anti-Inflammatory and Antibacterial Effects

The diverse structural makeup of alkaloid compounds in Papaveraceae plants significantly contributes to their notable anti-inflammatory and antibacterial properties. Among these compounds, benzophenanthridine alkaloids, protoberberine alkaloids, and aporphine alkaloids are particularly noteworthy. Sanguinarine (**2**) is a prominent compound within the class of benzophenanthridine alkaloids. The studies have shown that sanguinarine (**2**) could attenuate early stages of inflammation as well as reduce the expression of NF-κB and the subsequent production of pro-inflammatory cytokines to combat acetic acid-induced ulcerative colitis [121]. Another alkaloid chelerythrine (**3**) has been observed to inhibit the production of inflammatory factors such as TNF-α, IL-6, and IL-1β, which may provide protection acute lung injury and gastric ulcers. The mechanism might contribute to adjusting the inflammatory cytokine by regulating the NF-κB signaling pathway [122,123]. It has been found that dihydrochelerythrine (**10**), present in *Corydalis saxicola* Bunting, has a potent inhibitory effect on the hepatitis B virus [124]. Studies have demonstrated that berberine has the ability to exhibit anti-inflammatory properties by modulating a range of cellular physiological processes, such as cell cycle regulation, apoptosis, inflammatory pathways, and leukocyte migration [125]. Palmatine (**207**) demonstrated protective effects against inflammation and oxidative stress induced by monosodium uric acid through the modulation of the NF-κB/NLRP3 and Nrf2 pathways, as reported in a study [126]. Chelidonine (**1**), sanguinarine (**2**), and chelerythrine (**3**) possessed inhibiting effects against *E. coli*. Moreover, the presence of the medioxy group in the A ring of chelerythrine can improve antibacterial activity [127,128]. However, berberine (**196**) enhances the ability of antibiotics to kill bacteria through various mechanisms, such as reducing the expulsion of antibiotics from bacterial cells, preventing the formation of biofilms, and influencing the immune response and composition of gut bacteria in the host [129].

It is widely acknowledged that several genera in the poppy family possess anti-inflammatory and antibacterial properties. According to the findings of reference [130], *Corydalis baxicola bunting* has demonstrated significant inhibition of xylene-induced auricular swelling, acetic acid-induced increase in capillary permeability, and the formation of cotton ball granulomas in mice. Dai [131] reported that the total alkaloids found in *Corydalis saxicola bunting* have been suggested to exhibit therapeutic effects for acute hepatitis. The study indicated that *Yanhuanglian* suppositories have shown potential effectiveness in treating chronic pelvic inflammatory disease in rats. Furthermore, the total alkaloids in it have been shown to effectively inhibit HBsAg and HBeAg levels in HepG2.2.15 cells [124,132,133]. The high purity of the *Chelidonii herba* extract has been shown to inhibit several common bacteria, such as *Escherichia coli*, *Streptococcus*, *Pasteurella goldenseal*, *Staphylococcus aureus*, and *Salmonella* [123,134,135,136,137]. The study suggests that the alkaloids of ethanolic extract from *Herb* of *thinfruit hypecoum* have the potential to reduce lipopolysaccharide-induced inflammatory responses in mice by decreasing peripheral leukocyte exudation and reducing the expression of TNF-α and IL-6 in serum, effects comparable to those observed with cefaclor [138]. Furthermore, it has been discovered that total alkaloids found in *Corydalis bungeanae herba* may have anti-inflammatory properties by inhibiting the TLRs/NF-κB signaling pathway and reducing the secretion of inflammatory factors [139].

### 3.4. Anti-Tumor and Anti-Cancer Effects

In recent years, extensive research has focused on the potential anti-tumor and anti-cancer effects of plants from the opium poppy family. The primary anti-tumor effects are attributed to benzophenanthridine alkaloids and protoberberine alkaloids in Papaveraceae. Certain aporphine alkaloids and protopine alkaloids exhibit anti-cancer properties as well. Among them, benzophenanthridine alkaloids such as chelidonine (**1**), sanguinarine (**2**), and chelerythrine (**3**) may cause apoptosis by activating caspases, as well as cell induction of cycle arrest in cancer cells. Furthermore, chelerythrine (**3**) could induce autophagy in cancer cells. Benzophenanthridine alkaloids’ anti-cancer molecular mechanisms may be related to double-helical regions for binding and microtubule for binding [140,141,142,143,144,145,146]. Berberine (**196**), a representative compound of proberberine alkaloids, mediates apoptosis through mitogen-activated protein kinase. Moreover, berberine induces a halt in the G1 phase of the cell cycle by promoting the expression of (NSAID) activated gene-1 (NAG1) and activating transcription factor 3 (ATF3) in HCT116 cells. Berberine also enhances autophagy in glioblastoma by modulating the AMPK/mTOR/unc-51-like autophagy-activating kinase 1 (ULK1) pathway and inhibits the proliferation of human gastric cancer cells by deactivating the MAPK/mTOR/p70S6K/Akt signaling cascade both in vivo and in vitro. The molecular mechanisms underlying the anti-cancer properties of proberberine alkaloids involve the inhibition of enzyme activity and epigenetic modulation. The molecular mechanisms underlying the anti-cancer properties of proberberine alkaloids involve the inhibition of enzyme activity and epigenetic modulation. Berberine has been shown to downregulate nucleophosmin/B23, inhibit telomerase activity, and induce apoptosis in HL-60 cells. The observed reduction in cell proliferation in HepG2 cells following treatment with berberine was linked to the suppression of DNA methylation in the promoter regions of the cytochrome P450 2B6 (CYP2B6) and CYP3A4 genes [147,148,149,150,151]. Corydine from the Papaveraceae family inhibits CYP3A4, indicating a high-affinity interaction with this enzyme and demonstrating an anti-cancer effect [152]. Additionally, there have been reports indicating the potential anti-cancer properties of total alkaloids. One study revealed that the total alkaloids of *Corydalis saxicola bunting* inhibit the proliferation of human lung adenocarcinoma A549 cells and human tongue squamous carcinoma Tca8113 cells. This inhibition showed a positive correlation with both time and concentration. Additionally, aqueous extracts of *Corydalis saxicola bunting* suppressed the proliferation and migratory ability of HepG2 cells, primary hepatocellular carcinoma cells. As well, these alkaloid extracts up-regulated the expression of NF-κB p65 protein and mRNA [153]. Furthermore, *Meconopsis horridula* has been observed to inhibit cell proliferation and promote apoptosis in mouse lymphoblastic leukemia cell lines in a dose- and time-dependent manner [120]. These findings suggest that *Meconopsis horridula* holds potential therapeutic applications in cancer treatment. Additionally, *Hylomecon japonica (Thunb.) prantl et kundig*, and *Dicranostigma leptopodum* (Maxim.) Fedde have demonstrated anti-tumor effects by inhibiting the spread and metastasis of various tumors [39,51].

### 3.5. Protection of the Liver

Several total alkaloids or individual alkaloid components found in the Papaveraceae exhibit potent hepatoprotective properties. At present, protoberberine and protopine alkaloids have been extensively studied, with additional research also focusing on benzophenanthridine and benzylisoquinoline alkaloids. Berberine (**196**) has been shown to increase antioxidant levels in the liver through the activation of Nrf2/AREs, thus helping to regulate oxidative stress within the organ. Additionally, it can decrease inflammatory markers in the liver by blocking the MyD88/NF-κB pathway. Berberine also has the ability to suppress SREBP-1C expression, leading to a reduction in fat synthesis and the accumulation of lipid droplets in the liver, ultimately providing a protective effect against nonalcoholic fatty liver disease [154]. Dehydrocavidine (**234**), another proberberine alkaloid, has been shown to provide protection against CCl4-induced hepatic fibrosis in rats by decreasing oxidative stress, enhancing collagenolysis, and modulating genes associated with fibrosis [155]. Protopine (**466**) and allocryptopine (**467**) were found to have a significant impact on the mRNA levels of cytochrome oxidase P4501A in human hepatocytes and Hep G2 cells. However, there was no corresponding increase in P450 1A protein amount or viability levels [156]. Protopine (**466**) demonstrated the ability to suppress d-galactosamine-induced hepatitis by modulating a range of serum enzymes including SGPT, SGOT, ALP, and BL, as well as liver metabolites [157]. Research indicates that chelerythrine (**3**) has the potential to inhibit carbon tetrachloride-induced hepatic fibrosis in mice. Additionally, it has been observed to reduce blood levels of AST and ALT, improve the proliferation of intrahepatic tissues, alleviate acute and chronic liver injury, inhibit the proliferation of hepatic stellate cells, and reduce the synthesis of type I and type III collagen [136].

The total alkaloids of *Corydalis saxicola bunting* have been found to exhibit hepatoprotective and preventive effects against liver diseases, particularly in the areas of anti-hepatic injury, anti-hepatic fibrosis, anti-cholestasis, anti-fatty liver, and anti-viral hepatitis. Research suggests that the main mechanism of action is anti-lipid peroxidation [31,158]. One study suggests that the n-butanol extract from *Fructus Illcii Veri* may have a protective effect against liver injury caused by carbon tetrachloride in rats. Furthermore, it appears to have a significant impact on serum ghrelin and ghrelin [71]. The total alkaloid of *Artemisia annua* L. has been suggested to potentially offer protection against liver injury induced by carbon tetrachloride in mice. The alkaloid of *Meconopsis quintuplinervia reg*. showed a significant reduction in serum alanine aminotransferase, aspartate aminotransferase, lactate dehydrogenase activity, and malondialdehyde in the liver tissue homogenate of mice with immune liver injury, suggesting potential hepatoprotective effects [159].

### 3.6. Antitussive Effects and Other Pharmacological Effects

Studies have shown that the alkaloids of *poppy* husk, *Chelidonii herba* and *Hylomecon japonica (Thunb.) prantl et kundig*, are used to suppress cough and asthma [39,160,161]. The *Papaveraceae* of medicinal plants have various pharmacological effects, including immunomodulation, the regulation of intestinal flora [31], the attenuation of kidney damage, the treatment of osteoporosis [26], the treatment of leukemia [51], and the treatment of lupus erythematosus, and they act as a urease inhibitor [156]; have antispasmodic [17,162], anti-obesity, and anti-diabetic effects; treat varicose veins [38]; relax smooth muscles [162]; and promote animal growth [50].

## 4. Conclusions and Prospects

The opium poppy encompasses numerous species, many of which hold significance in Tibetan and Mongolian medicinal practices. Certain varieties, such as poppy and yanhuisuo, boast a rich history of medicinal usage. The primary constituents of the opium poppy are isoquinoline alkaloids.

Firstly, this paper represents the first comprehensive compilation of over 500 alkaloid components reported within the *Papaveraceae* family. Corresponding structures are delineated to serve as a reference for future studies. Existing data indicate that the *Papaveraceae* family harbors the majority of isoquinoline alkaloids, constituting approximately 89% of the total alkaloids. The discovery of these compounds lays the groundwork for further exploration into their pharmacodynamic and pharmacological effects.

Secondly, this paper reviews the pharmacological effects of plant alkaloids found in *Papaveraceae*. Previous studies have identified several significant effects, including analgesic properties, impacts on the cardiovascular system, anti-inflammatory and anti-cancer activities, as well as liver protection. These effects have been elucidated through both in vitro and in vivo studies. However, there remains a dearth of research on their effects on cough and asthma, which necessitates further investigation. Moreover, studies indicate that medicinal plants from the *Papaveraceae* family possess immunoregulatory properties, influence intestinal flora, exhibit nephroprotective effects, and show potential in treating conditions such as osteoporosis, leukemia, lupus erythematosus, and uremia.

Future research on poppy plants can focus on several aspects. Regarding plant diversity, *Papaveraceae* encompasses numerous widely distributed species, yet few species have been thoroughly studied, with a significant concentration on *Corydalis.* Therefore, to fully realize the medicinal potential of *Papaveraceae* plants, further studies on their chemical composition, including other compounds, are warranted. From a pharmacological perspective, although various effects have been identified, many studies rely on crude plant extracts. Research on individual compounds remains limited, lacking in-depth molecular-level investigation, which impedes the effective summarization of structure–activity relationships. The extraction and detailed molecular-level analysis of alkaloids from plants of the *Papaveraceae* family present numerous challenges in the field. Future research efforts should focus on investigating the medicinal mechanisms and bioavailability of various alkaloids. Simultaneously, attention should be given to other plant components.

## Data Availability

The original contributions presented in the study are included in the article (and Supplementary Material), further inquiries can be directed to the corresponding authors.

## Supplementary Materials

The following supporting information can be downloaded at https://www.mdpi.com/article/10.3390/molecules29163778/s1, Table S1: Alkaloids isolated from *Papaveraceae*. Figures S1–S9: Alkaloids isolated from *Papaveraceae*.
