# Supplementary material for: Recent Advances in Alkaloids from Papaveraceae in China: Structural Characteristics and Pharmacological Effects
Source: Molecules. 2024 Aug 9;29(16):3778. doi: 10.3390/molecules29163778 (PMC11357172; doi:10.3390/molecules29163778)
Supplement: Supplementary file 1 [file molecules-29-03778-s001.zip › supplementary figures.docx]

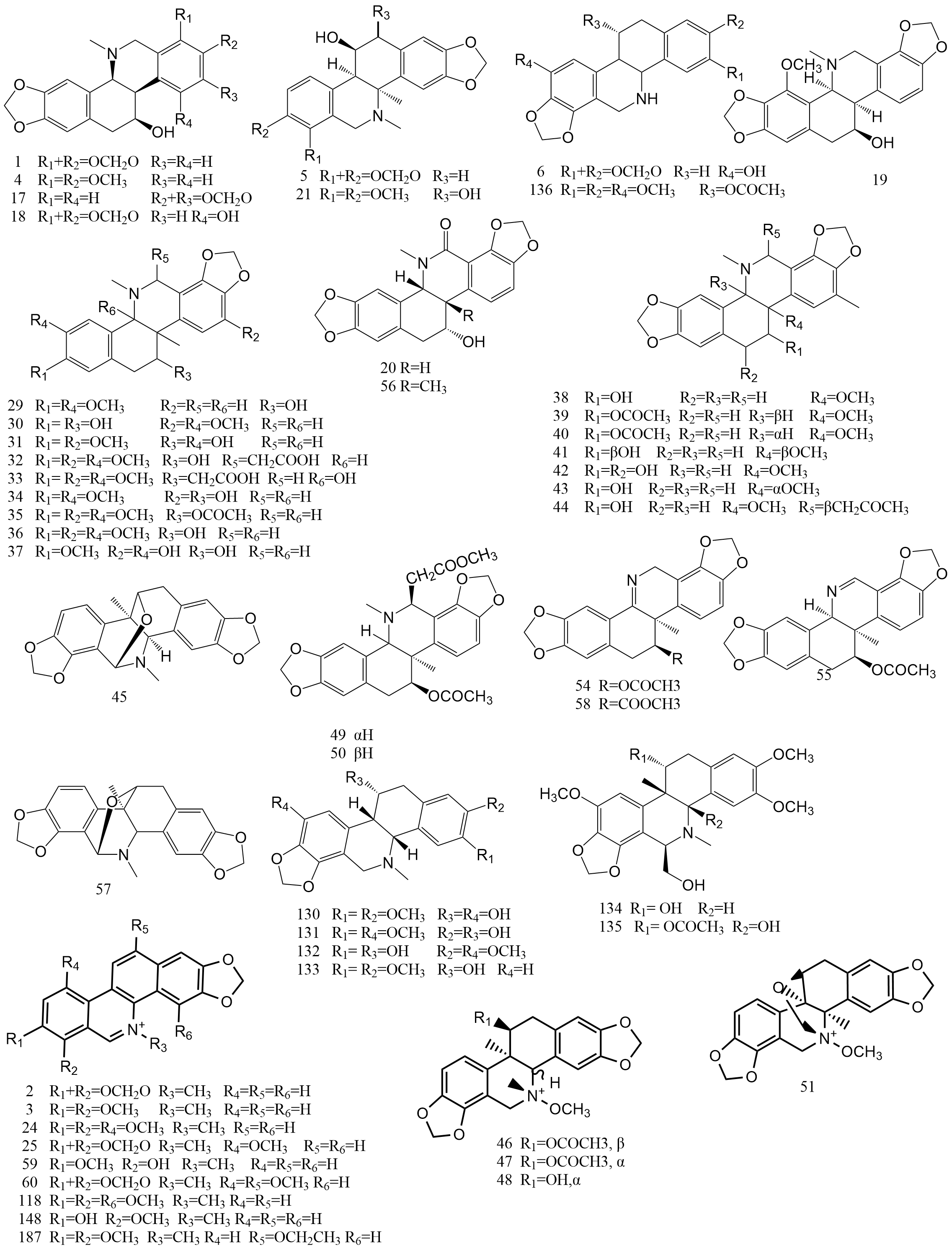


Figure S1. Benzophenanthridine alkaloids-1.


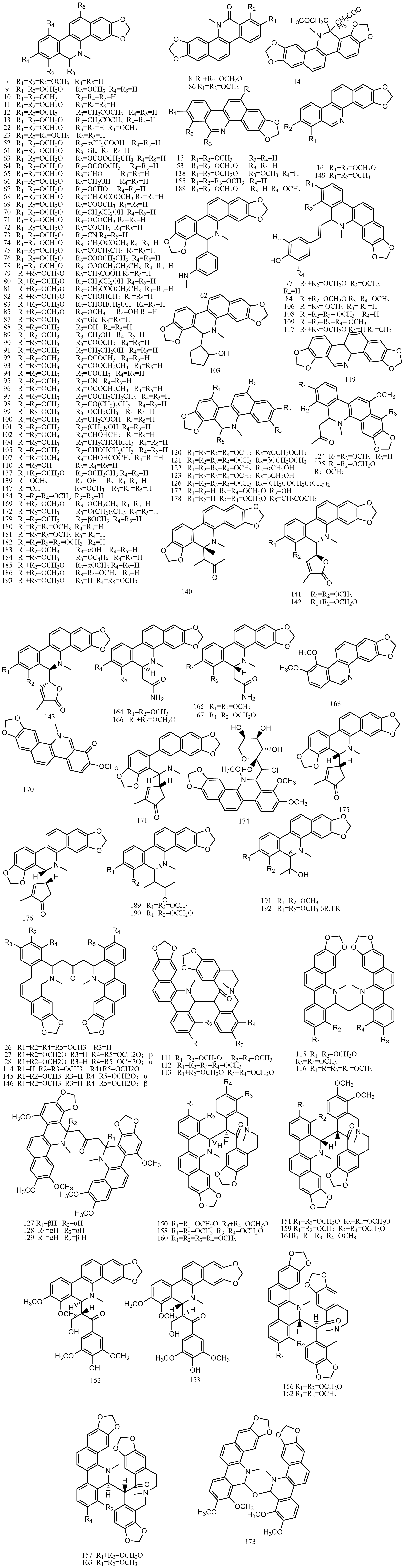


Figure S2. Benzophenanthridine alkaloids-2.


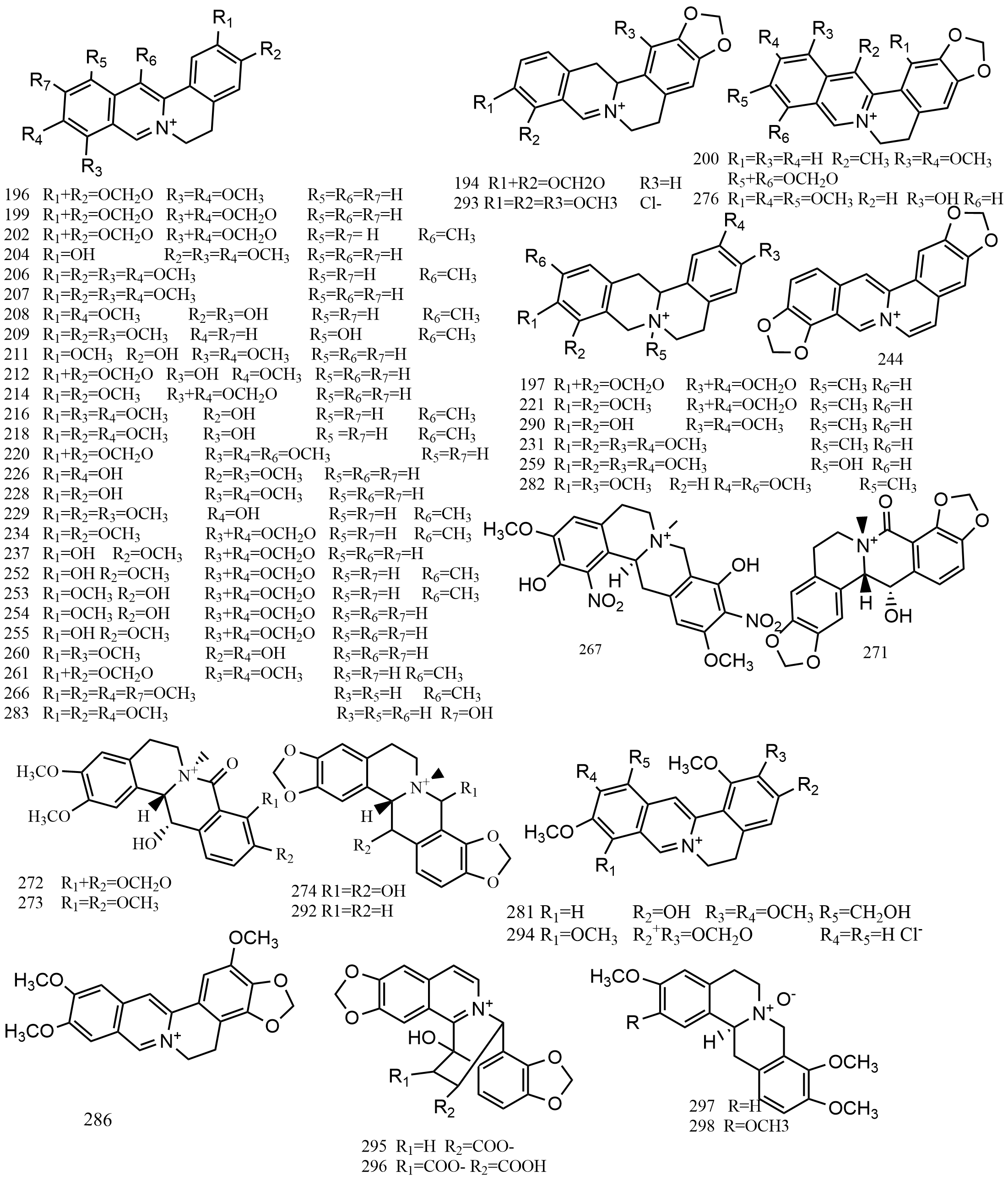


Figure S3. Protoberberine alkaloids-1.


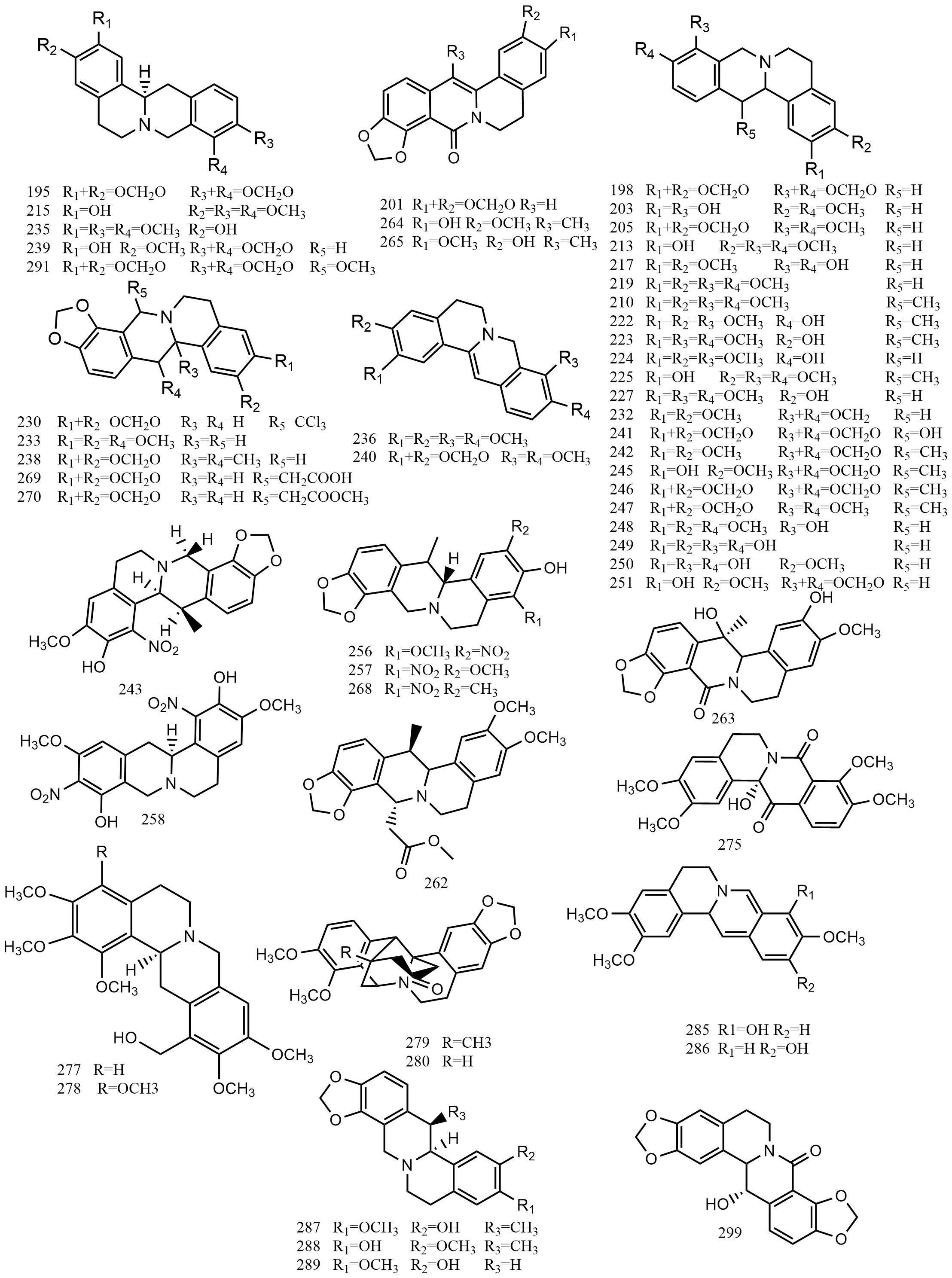


Figure S4. Protoberberine alkaloids-2.


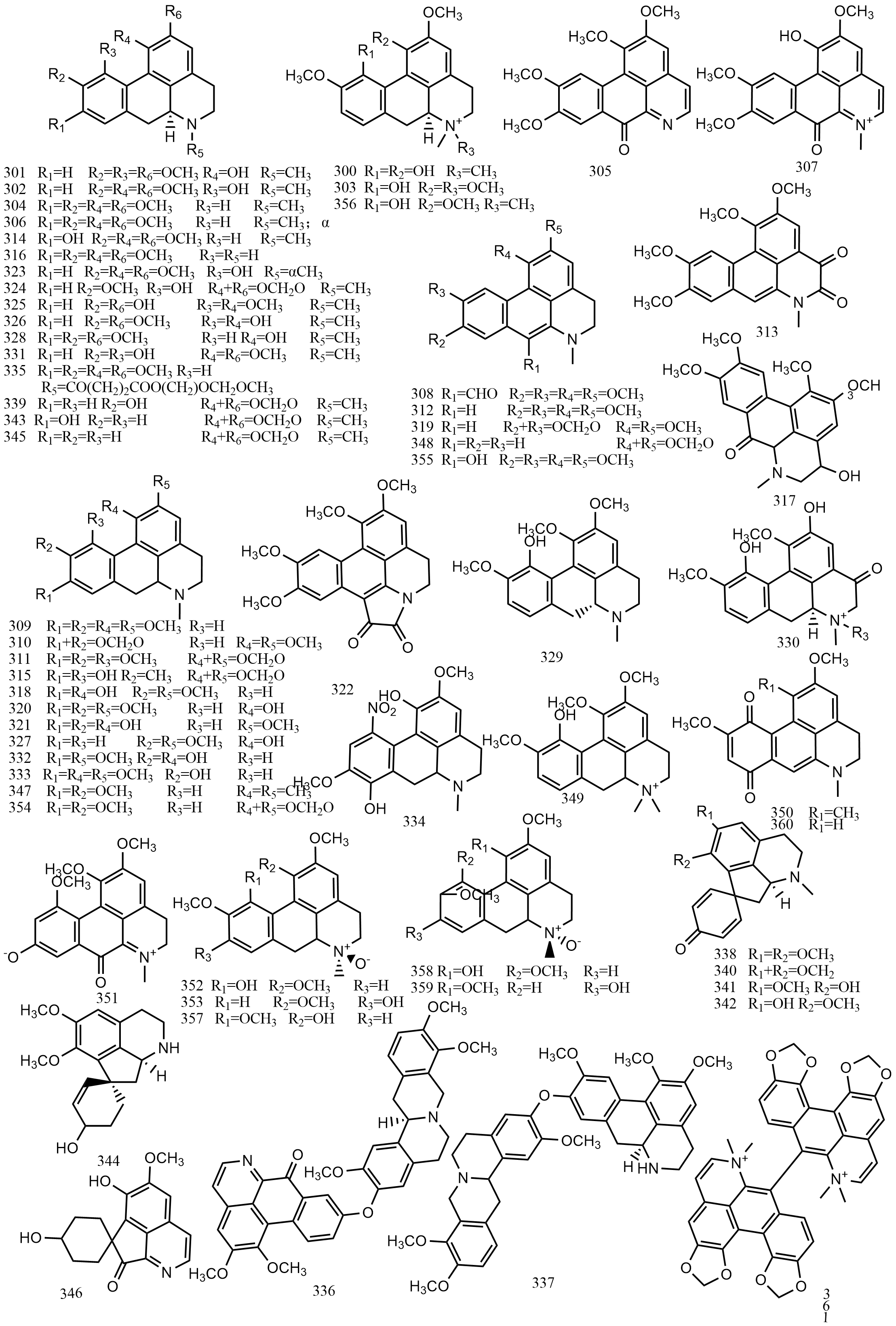


Figure S5. Aporphine alkaloids.


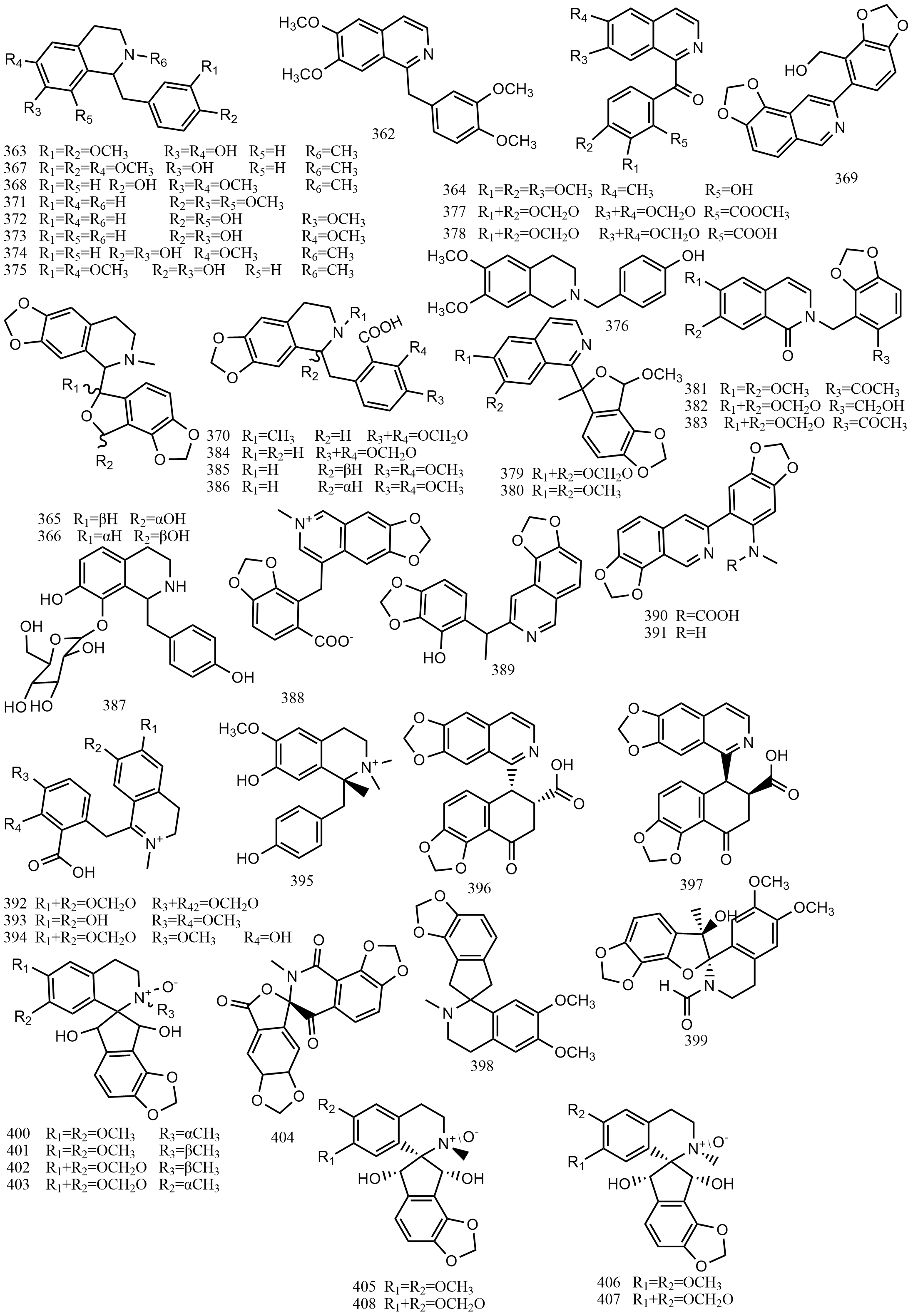


Figure S6. Benzylisoquinoline alkaloids.


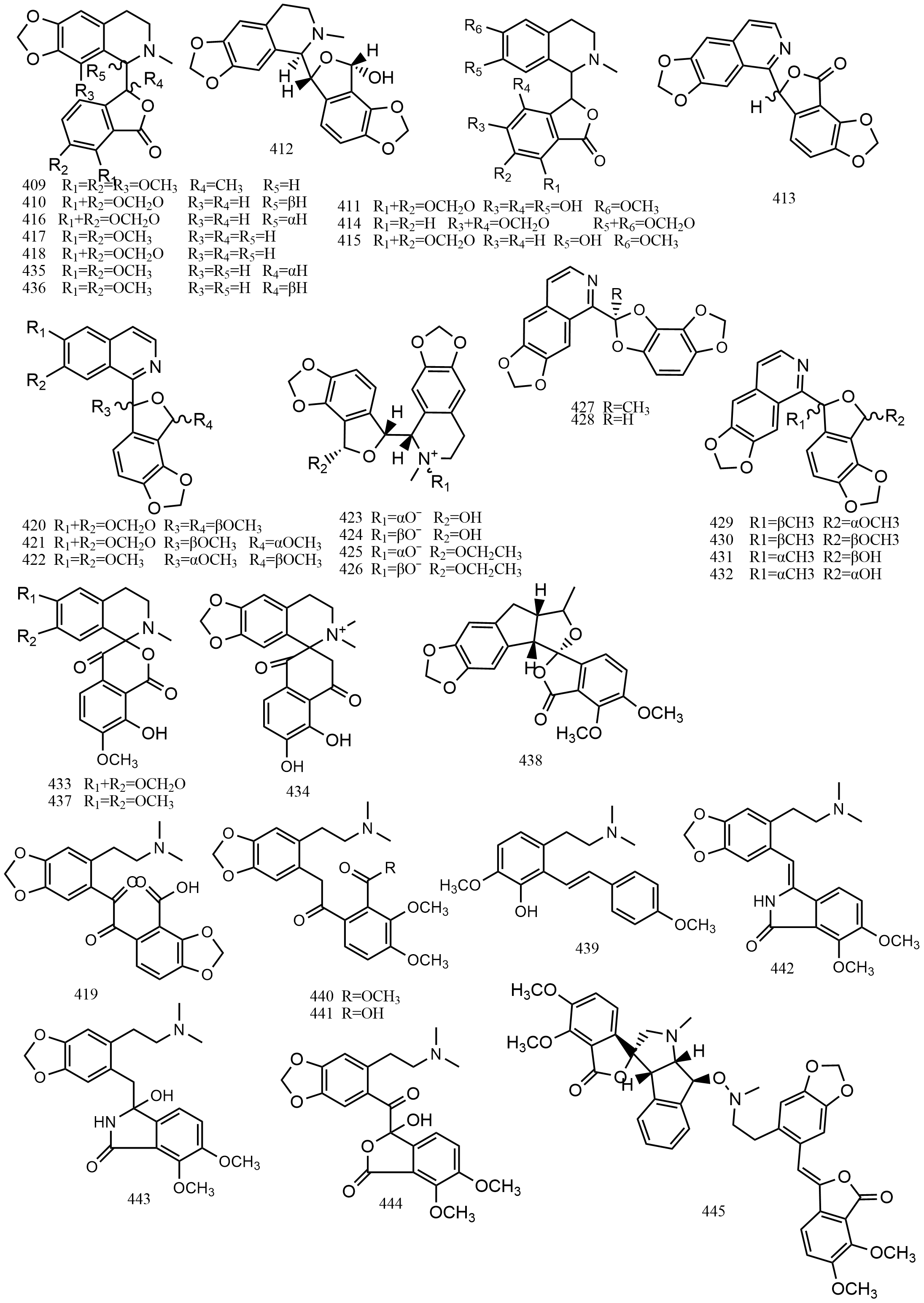


Figure S7. Benphthaleoquinoline alkaloids.


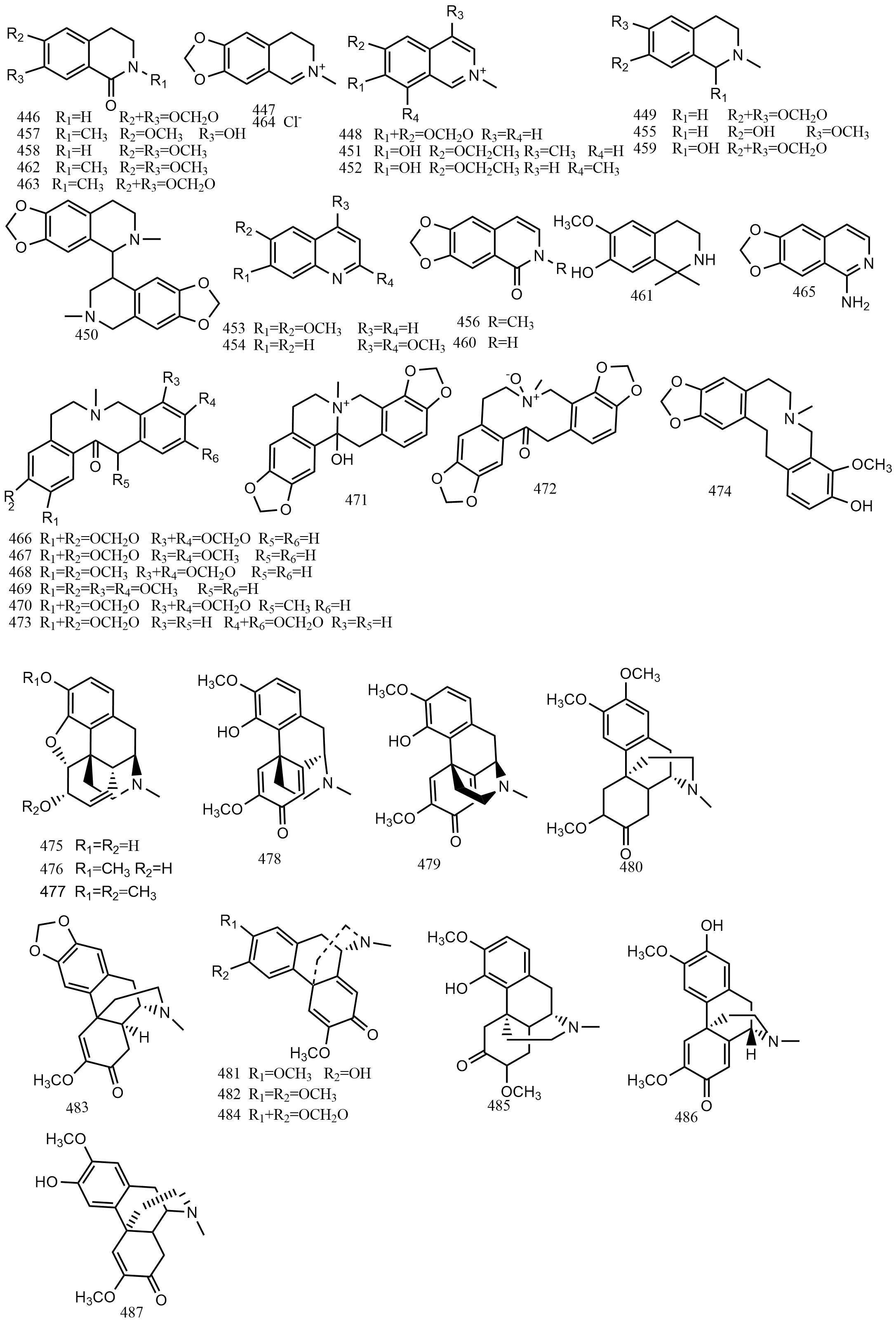


Figure S8. Simple-isoquinoline, Protopine, Morphine alkaloids.


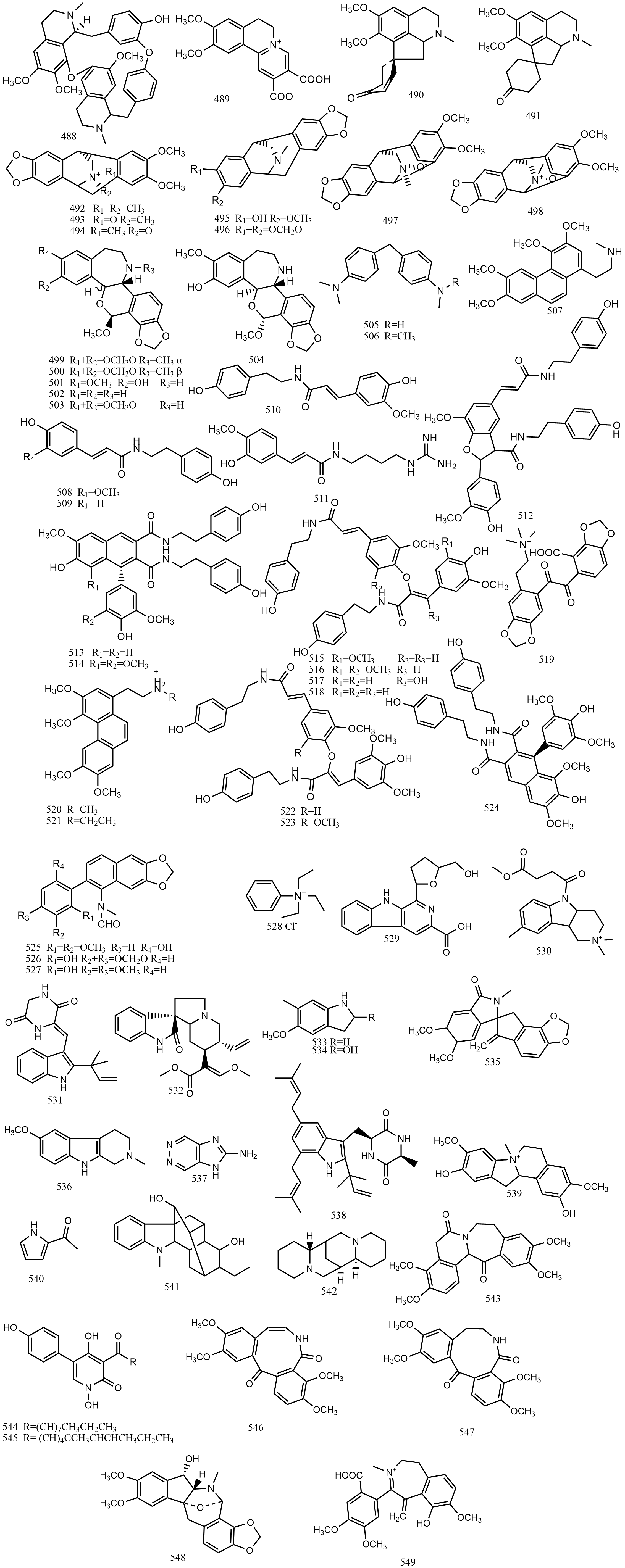


Figure S9. Others alkaloids.
